# Systematic Analysis and Validation of the Prognosis, Immunological Role and Biology Function of the Ferroptosis-Related lncRNA GSEC/miRNA-101-3p/CISD1 Axis in Lung Adenocarcinoma

**DOI:** 10.3389/fmolb.2021.793732

**Published:** 2022-03-07

**Authors:** Xiulin Jiang, Yixiao Yuan, Lin Tang, Juan Wang, Dahang Zhang, Lincan Duan

**Affiliations:** ^1^ The Department of Thoracic Surgery, The Third Affiliated Hospital of Kunming Medical University (Yunnan Tumor Hospital), Kunming, China; ^2^ Key Laboratory of Animal Models and Human Disease Mechanisms of Chinese Academy of Sciences, Kunming Institute of Zoology, Kunming, China

**Keywords:** NSCLC, ferroptosis, ceRNA, immune cell infiltration, cell proliferation, cell migration

## Abstract

Lung adenocarcinoma (LUAD) is the most common type of lung cancer, accounting for approximately 85% of pulmonary malignancies. Emerging evidence has demonstrated that ferroptosis plays a central role in both immunities as well as tumor proliferation. However, the clinical significance, immunological function, and upstream modulatory mechanism of ferroptosis-related genes in LUAD remain unclear. Here, we utilized various bioinformatics data to identify differentially expressed (DEGs) and prognosis-related ferroptosis (FRGs) genes in LUAD. Based upon identified DEGs, FRG, and ceRNA modulatory networks were constructed. Pearson’s correlation analysis was used to evaluate the correlation between FRGs and the tumor mutational burden, microsatellite instability, tumor-infiltrating immunity, cellular checkpoint control, and drug sensitivity in LUAD. A loss-of-function analysis was performed to verify the function of CISD1 in LUAD progression. Our findings revealed that certain FRGs (CISD1, ATP5MC3, PGD, SLC7A11, ACSL3, and FANCD2) are significantly upregulated in LUAD and that their elevated expression is associated with both advanced tumor stage and unfavorable prognosis. Furthermore, Kyoto Encyclopedia of Genes and Genomes (KEGG) enrichment results revealed these FRGs to be primarily involved in ferroptosis and glutathione metabolism in LUAD. We constructed a prognostic FRG-based model capable of accurately predicting LUAD patient overall survival with high specificity. The upstream lncRNA GSEC/miRNA-101-3p regulatory axis involving CISD1, ATP5MC3, and PGD was identified to be relevant in tumor progression. We also found GSEC, CISD1, ATP5MC3, and PGD to be upregulated, with miRNA-101-3p downregulated, in the setting of LUAD. Immunohistochemical analysis revealed CISD1, ATP5MC3, and PGD overexpression in LUAD tissue samples; CISD1 knockdown was noted to significantly inhibit LUAD proliferation and migration. In summary, this study characterizes relevant functional roles of the lncRNA GSEC/miR-101-3p axis in the setting of LUAD and suggests diagnostic and therapeutic biomarkers potentially useful in the clinical management of this illness.

## Introduction

Lung cancer remains the commonest fatal condition globally, with an estimated 2.09 million new cases and 1.76 million deaths annually (Rivera and Wakelee, 2016). Non-small cell lung cancer, the most frequently encountered disease subtype, typically results in adverse clinical outcomes and carries a 5-year survival rate of only 18% (Schwartz and Cote, 2016). Despite advances in early detection and treatment standardization, management strategies remain varied and range from chemo-radiotherapy to immunotherapy. Urgent identification of novel, specific biomarkers with clinicopathological and prognostic significance is thus necessary for developing successful lung adenocarcinoma (LUAD) management strategies.

Ferroptosis describes a recently discovered type of programmed cell death distinct from apoptosis that is iron-dependent and characterized by both lipid peroxidation and reactive oxygen species production (Wang et al., 2019; Luo et al., 2021; Xu et al., 2021). Ferroptosis has been reported to play an essential role in lung cancer progression; the interaction of LINC00336 with ELAVL1 was found to result in the inhibition of ferroptosis in LUAD (Wang et al., 2019). Similarly, the interaction of G3BP1 with lncRNA-P53RRA was reported to facilitate both ferroptosis and apoptosis in lung cancer *via* regulation of p53 expression (Mao et al., 2018). Ferroptosis was additionally reported to be regulated *via* cellular redox and cell cycle signaling pathways (Yang and Stockwell, 2016). Prior studies have suggested several oncogenic signaling pathways related to the ferroptosis process to inhibit tumor progression *via* modulation of ferroptosis (Kang et al., 2019; Mou et al., 2019). For instance, P53, a well-studied tumor-suppressor gene, was reported to suppress cystine/glutamate antiporter expression and thus inhibit the ferroptosis process (Mou et al., 2019). However, a comprehensive analysis has yet to establish the prognostic value of ferroptosis-related genes (FRGs) and their relevant upstream regulatory axis in LUAD.

Analysis of gene expression, mutation, DNA methylation, and prognostic value in LUAD revealed CISD1, ATP5MC3, PGD, SLC7A11, ACSL3, and FANCD2 to be significantly upregulated in LUAD and elevated expression of these FRGs to be associated with advanced tumor stage and poor prognosis. Furthermore, KEGG enrichment findings revealed these FRGs to primarily be involved in both ferroptosis and glutathione metabolism in the setting of LUAD. Six prognosis-related genes were also used to construct an FRG-based prognostic model capable of accurately predicting LUAD patient overall survival with high specificity. Finally, CISD1, ATP5MC3, PGD, SLC7A11, ACSL3, and FANCD2 expression was confirmed to significantly correlate with tumor mutational burden (TMB), microsatellite instability (MSI), immune cell infiltration, cellular checkpoint dysfunction, and cancer sensitivity to drugs. Additionally, we identified the upstream regulatory (namely, the lncRNA GSEC/miRNA-101-3p) axis of relevant FRGs (CISD1, ATP5MC3, PGD) and found GSEC, CISD1, ATP5MC3, and PGD to be upregulated, with miRNA-101-3p downregulated, in the setting of LUAD. Knockdown of CISD1 was noted to significantly inhibit the proliferative and migratory capabilities of LUAD cells. In summary, by characterizing the functional roles of the lncRNA GSEC/miR-101-3p axis in LUAD, we suggest diagnostic and therapeutic biomarkers potentially useful in the future clinical management of this condition.

## Materials and Methods

### Data Collection

The Cancer Genome Atlas (TCGA)-LUAD cohort data and corresponding clinical information of 535 LUAD patients were downloaded from the TCGA website up to November 14, 2020 (https://portal.gdc.cancer.gov/repository). The gene expression profiles were normalized using the scale method provided in the “limma” R package. Data analysis was performed with the R (version 3.6.3) and ggplot2 (3.3.3) packages. The expression data were normalized to transcripts per kilobase million (TPM) values before further analysis.

### Identification of Differentially Expressed 34 FRGs

A total of 34 FRGs were obtained from previous reviews (Su et al., 2020; Feng et al., 2020), which are shown in Supplementary Table S1. The difference in FRG expression in LUAD and normal tissues was identified using the “limma” packages. We then constructed a gene–gene interaction network for 34 FRGs using the GeneMANIA (http://www.genemania.org) database (Warde-Farley et al., 2010).

### Gene Mutation Analysis of FRGs

The mutation frequency and oncoplot waterfall plot of 34 FRGs in LUAD patients were analyzed by the Gene Set Cancer Analysis (http://bioinfo.life.hust.edu.cn/web/GSCALite/) database (Liu et al., 2018).

### Functional Enrichment Analysis

Gene Ontology (GO), consisting of the biological process (BP), cellular component (CC), and molecular function (MF) categories, was conducted with the “ggplot2” package in R software. Similarly, this package was also utilized to perform Kyoto Encyclopedia of Genes and Genomes (KEGG) analysis (Ito and Murphy, 2013).

### Development of the Ferroptosis-Related Gene Prognostic Model

Development of the FRG prognostic model was performed as documented (Lin et al., 2021). Briefly, we performed the Cox regression analysis to examine the prognostic significance of the FRGs. For Kaplan–Meier curves, *p*-values and hazard ratios (HRs) with 95% confidence intervals (CIs) were generated by log-rank tests and univariate Cox proportional hazard regression. FRGs with a significant prognostic value was selected for further analysis. Based on these prognostic FRGs, LASSO Cox regression analysis was then used to construct the prognostic model. The TCGA LUAD patients were divided into low- and high-risk subgroups according to the median risk score, and the overall survival time was compared between the two subgroups *via* Kaplan–Meier analysis. The predictive accuracy of each gene and the risk score was evaluated by performing time receiver-operating characteristic (ROC) analysis. Considering the clinical characteristics, a predicted nomogram was developed to predict the 1-, 3-, and 5-year overall survival. A forest was used to show the *p*-value, HR, and 95% CI of each variable through the “forest plot” R package.

### Prediction of lncRNA and ceRNA Network Construction

We used starbase (http://starbase.sysu.edu.cn/) to predict the potential upstream miRNAs of FRGs and examine the expression, prognosis, and correlation between miRNA-101-3p and lncRNA, as well as to predict the binding sites among miRNA, mRNA, and lncRNA (Li et al., 2014). We utilized lncLocator (www.csbio.sjtu.edu.cn/bioinf/lncLocator.) and CPC2 (http://cpc2.cbi.pku.edu.cn) to explore the subcellular localization and the protein-coding ability of lncRNAs (Kang et al., 2017; Cao et al., 2018).

### Analysis of the Immunological Roles of FRGs in Lung Cancer

We utilize TIMER (https://cistrome.shinyapps.io/timer/) and XCELL tools (https://xcell.ucsf.edu/) to examine the immunological roles of FRGs (Aran et al., 2017; Li et al., 2017), including the correlation between diverse immune cells and immune regulators. The TMB and MSI scores were obtained from TCGA. A correlation analysis between the FRG expression and TMB or MSI was performed using Spearman’s method.

### Analysis of the Correlation Between the FRGs Expression and Drug Sensitivity

We utilize the Genomics of Drug Sensitivity in Cancer (GDSC) (www.cancerRxgene.org) and the Cancer Therapeutics Response Portal (CTRP) databases to analyze the correlation between FRG expression and drug sensitivity (Basu et al., 2013; Yang et al., 2013).

### Cells and Cell Culture Conditions

The BEAS-2B cell line was purchased from the cell bank of Kunming Institute of Zoology and cultured in BEGM media (Lonza, Morrisville, NC, USA, CC-3170). Lung cancer cell lines, including A549 and H1975, were purchased from Cobioer (Nanjing, China), with the STR document; A549 and H1975 cells were all cultured in RPMI 1640 medium (Corning, Tewksbury, MA, USA) supplemented with 10% fetal bovine serum (Cat# 10099141C, Gibco, Grand Island, NY, USA) and 1% penicillin/streptomycin.

### Quantitative Real-Time PCR

The qRT-PCR assay was performed as documented (Jiang et al., 2018). Total RNA was extracted according to the manufacturer’s protocol, and then reverse-transcribed using RT Reagent Kit (Takara Bio, Beijing, China, Cat# RR047A; Tiangen Biotech, Beijing, China, Cat# KR211-02). Real-time PCR was performed by FastStart Universal SYBR Green Master Mix (Roche, Cat# 04194194001; Tiangen Biotech, Beijing, China, Cat# FP411-02) using an Applied Biosystems 7500 machine. The primer sequences are listed as follows: GSEC-F: TTC​CAA​TTA​ACC​TGG​CCG​GAG, GSEC-R: GTC​AGC​CAA​CCC​ATT​GCA​AC, PGD-F: ATG​GCC​CAA​GCT​GAC​ATC​G, PGD-R: AAA​GCC​GTG​GTC​ATT​CAT​GTT, CISD1-F: CTG​ACT​TCC​AGT​TCC​AGC​GT, CISD1-R:TGATCAGAGGGCCCACATTG, ATP5G3-F: ATG​TTC​GCC​TGC​GCC​AAG, ATP5G3-R: GGC​AAA​CAA​GAT​CAA​GAA​CGC​A, miRNA-101-3p: TAC​AGT​ACT​GTG​ATA​ACT​GAA. β-Actin was used to normalize expression levels: β-actin-F: CTTCGCGGGCGACGAT, β-actin-R: CCA​TAG​GAA​TCC​TTC​TGA​CC. The expression quantification was obtained with the 2−ΔΔCt method.

### Cell Proliferation and Cell Migration Assays

Cell proliferation assay was performed as previously described (Xu et al., 2020). Briefly, the indicated cells were plated onto 12-well plates, and the cell numbers were subsequently counted each day using an automated cell analyzer Countstar (Shanghai Ruiyu Biotech Co., Shanghai, China, IC1000). Cell migration assay was performed as previously described (Xiong et al., 2021). For transwell assay, 1-2×104 cells in 100 l serum-free medium were plated in an 8.0-µm, 24-well plate chamber insert (Corning Life Sciences, catalog no. 3422), with a medium containing 10% FBS at the bottom of the insert. Cells were incubated for 24 h and then fixed with 4% paraformaldehyde for 20 min. After washing, cells were stained with 0.5% crystal violet-blue. The positively stained cells were examined under the microscope.

### Immunohistochemistry Staining

The immunohistochemistry staining assay was performed as documented (Dixon et al., 2012). Briefly, cancer tissues were collected; the primary antibody was incubated overnight and the second antibody incubated. Finally, the instrument was developed. Detailed information of the primers used in this study is as follows: PGD, Catalog number: #13389, dilution, 1:100, CST, Shanghai, China; CISD1, Catalog number: #83775, dilution, 1:200, CST, Shanghai, China; and ATP5MC3, Catalog number: ab129742, dilution, 1:100, Abcam, Shanghai, China.

### Statistical Analysis

Analysis of the FRG expression lung cancer was estimated using t-tests. For survival analysis, the HR and *p*-value were calculated employing univariate Cox regression analysis. Kaplan–Meier analysis was used to examine the survival time of patients stratified according to high or low levels of the FRG expression. *p*-values less than 0.05 were considered statistically significant. For all figures, ∗, ∗∗, and ∗∗∗ indicate *p* < 0.05, *p* < 0.01, and *p* < 0.001, respectively.

## Results

### Analysis of FRG Expression and Mutation in LUAD

**FIGURE 1:**
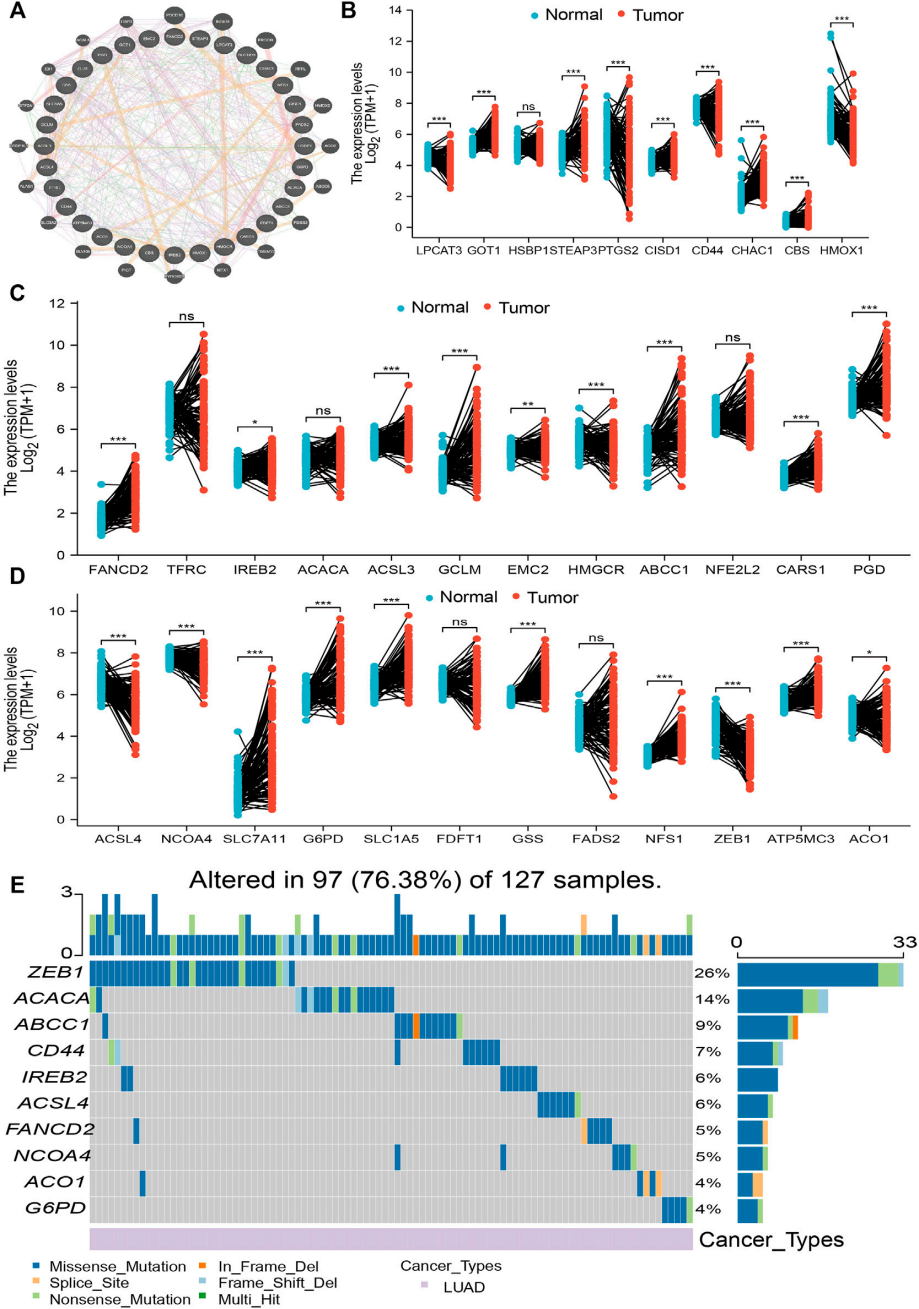
Analysis of the expression and gene mutation of FRGs in LUAD. **(A)** The gene–gene interaction networks of FRG analysis by the GeneMANIA database. **(B–D)** The expression of FRGs in LUAD is examined by the TCGA database. **(E)** The mutation frequency and classification of FRGs in LUAD were examined by GSCA tools. For all figures, ∗, ∗∗, and ∗∗∗ indicate *p* < 0.05, *p* < 0.01, and *p* < 0.001, respectively.

The expression of 34 FRGs and their function in the setting of LUAD was analyzed using data obtained from the TCGA database (Supplementary Table S1). Findings confirmed 26 genes to be upregulated and seven genes to be downregulated in lung cancer; no other significant differences were noted (Figures 1A–D). Furthermore, analysis of FRG mutations in LUAD revealed 97 of 127 (76.38%) samples to exhibit genetic mutations; *ZEB1* exhibited the highest mutation frequency and was followed by *ACACA* and *ABCC1* (Figure 1E). Finally, analysis of correlations among copy number variation (CNV), DNA methylation, and FRG expression in LUAD revealed FDFT1, IREB2, NFS1, TFRC, HSBP1, ACO1, LPCAT3, CARS, GSS, ACACA, ACSL3, CISD1, SLC1A5, and NCOA4 expression to positively correlate with their respective CNV (Supplementary Figure S1A). Meanwhile, DNA methylation was found to negatively associate with CBS, FDFT1, STEAP3, FADS2, SCL1A5, GCLM, GOT1, SLC7A11, CD44, ACSL3, and G6PD expression in LUAD (Supplementary Figure S1B). Collectively, these data indicate that CNV and DNA methylation exert a critical influence on FRG expression in LUAD.

### Analysis of FRG Function in LUAD

**FIGURE 2:**
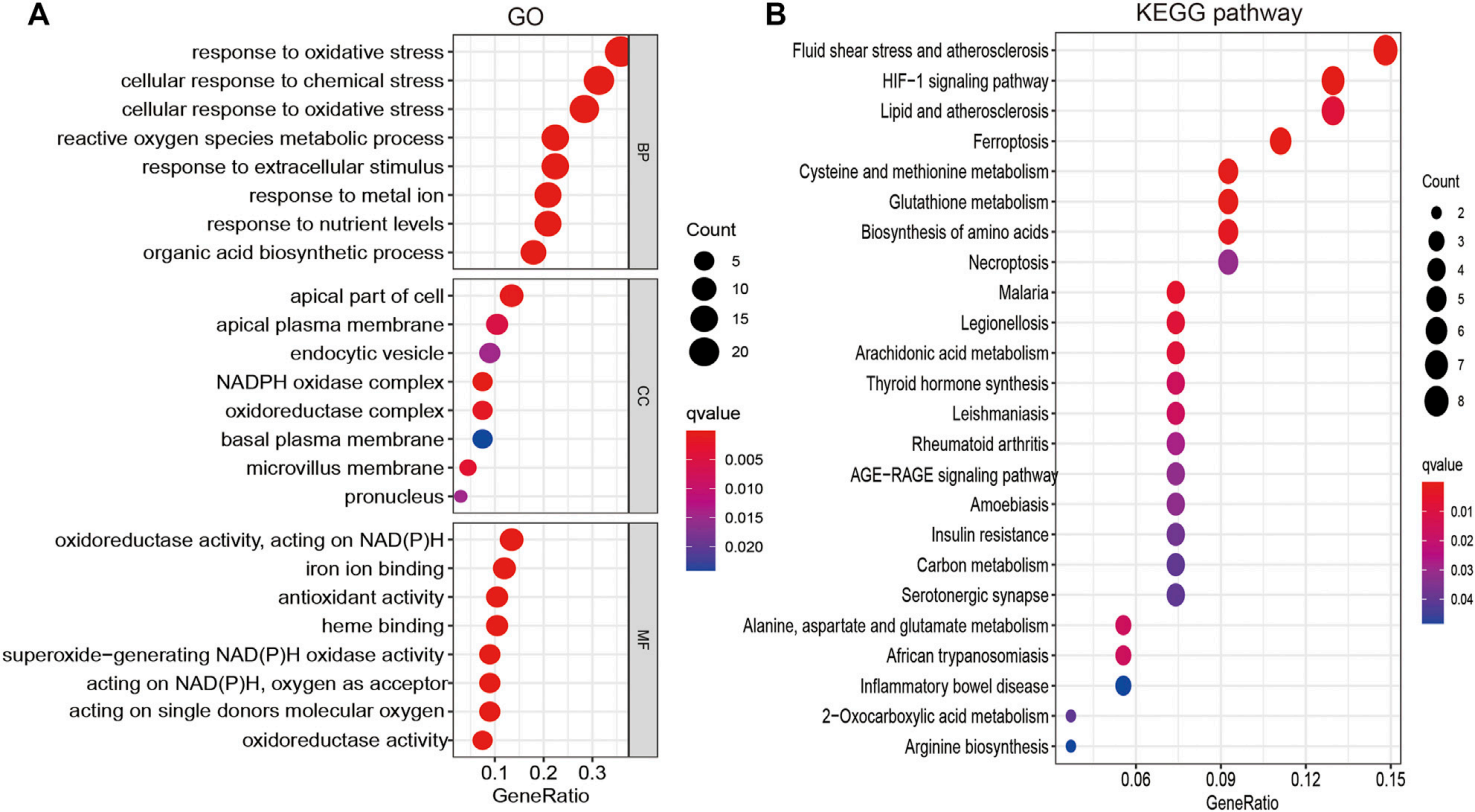
Analysis of the functions of FRGs in LUAD. **(A)** The biological process involved by FRGs in LUAD. **(B)** The KEGG pathway is involved by FRGs in LUAD.

To explore the function of FRGs in LUAD, GO and KEGG enrichment analyses were performed. Results revealed 34 FRGs to be primarily involved in cellular responses to oxidative and other chemical stress, reactive oxygen species metabolism, extracellular stimuli, nutrient levels, biosynthetic processes, and iron ion binding (Figure 2A). Furthermore, KEGG pathway enrichment analysis revealed these FRGs to be primarily involved in vascular fluid dynamics and pathogenesis of atherosclerosis, HIF-1 signaling, lipid metabolism, ferroptosis, amino acid metabolism, necroptosis, thyroid hormone synthesis, serotonergic communication, and inflammatory bowel disease pathogenesis, as well as the metabolism of arachidonic acid, aspartate, alanine, glutamate, and 2-monocarboxylic acid (Figure 2B). Collectively, these data confirm that FRGs influence cancer cell metabolism, with ferroptosis playing an essential role in LUAD progression.

### The Prognostic Value of FRGs in LUAD

**FIGURE 3:**
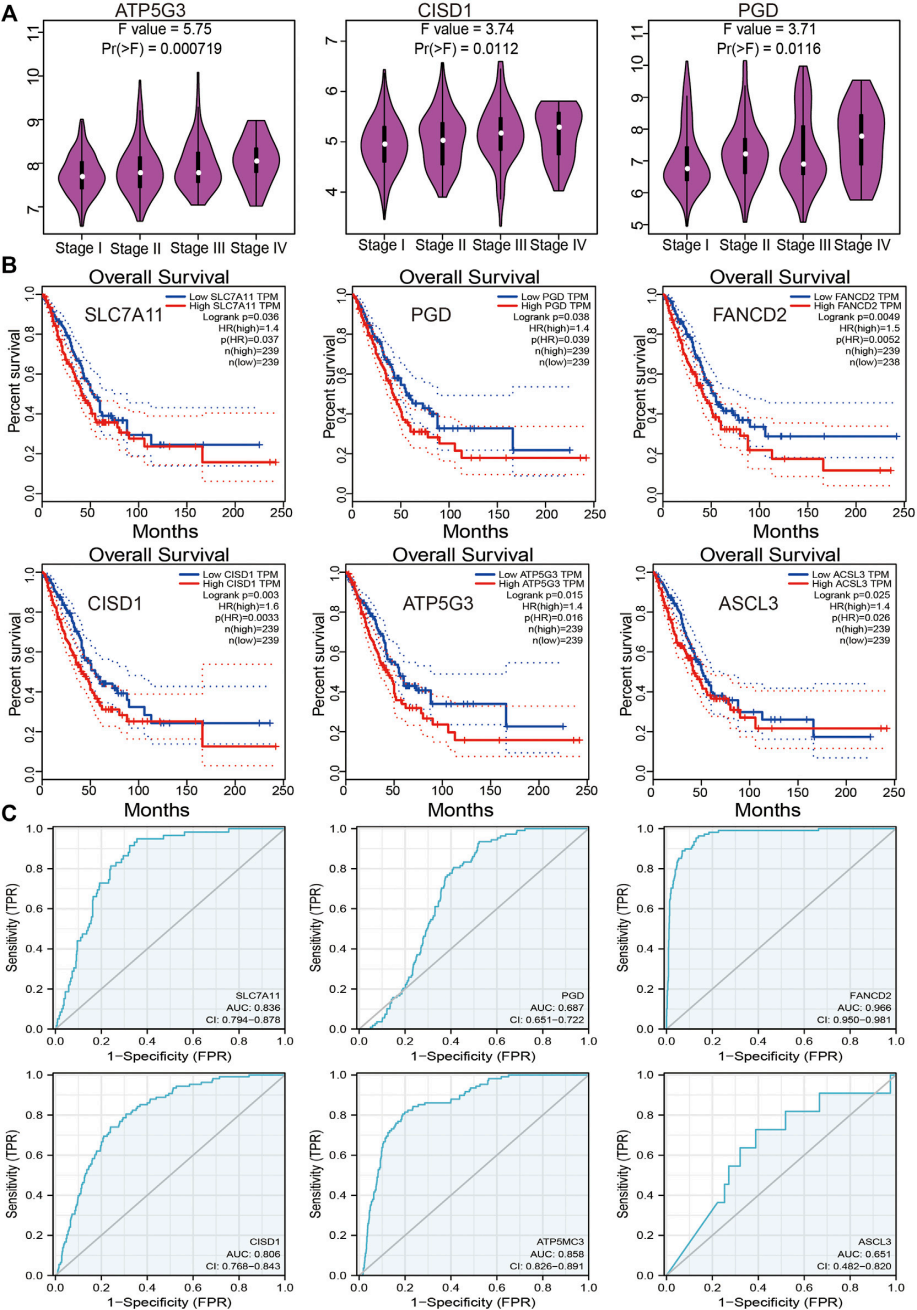
Analysis of the prognosis of FRGs in LUAD. **(A)** The pathologic stage of FRGs in LUAD is examined by the GEPIA database. **(B)** The overall survival of FRGs in LUAD was examined by the GEPIA database. **(C)** The ROC curve of FRGs in LUAD is examined by the TCGA database.

An investigation of the correlation between FRG expression and tumor stage revealed only ATP5G3, CISD1, and PGD expression to positively correlate with the LUAD stage (Figure 3A). Evaluation of the prognostic value of FRGs revealed elevated SLC7A11, PGD, FANCD2, CISD1, ATP5G3, and ASCL3 expression to closely correlate with adverse clinical outcomes in LUAD (Figure 3B). The diagnostic value of FRGs was evaluated using ROC analysis. Findings confirmed SLC7A11, FANCD2, CISD1, and ATP5G3 expression to accurately predict the LUAD prognosis area under the curve (AUC > 0.8) (Figure 3C). Higher levels of SLC7A11, FANCD2, SIDS1, ATP5G3, and ASCL3 expression were similarly found to correlate with disease-specific survival in LUAD (Supplementary Figure S2A). Elevated SLC7A11, ATP5G3, and ASCL3 expressions were found to correlate with progression-free survival in LUAD (Supplementary Figure S2B). Our findings thus confirm that FRG plays an essential role in LUAD progression.

### Construction of an FRG-Based Prognostic Model

**FIGURE 4:**
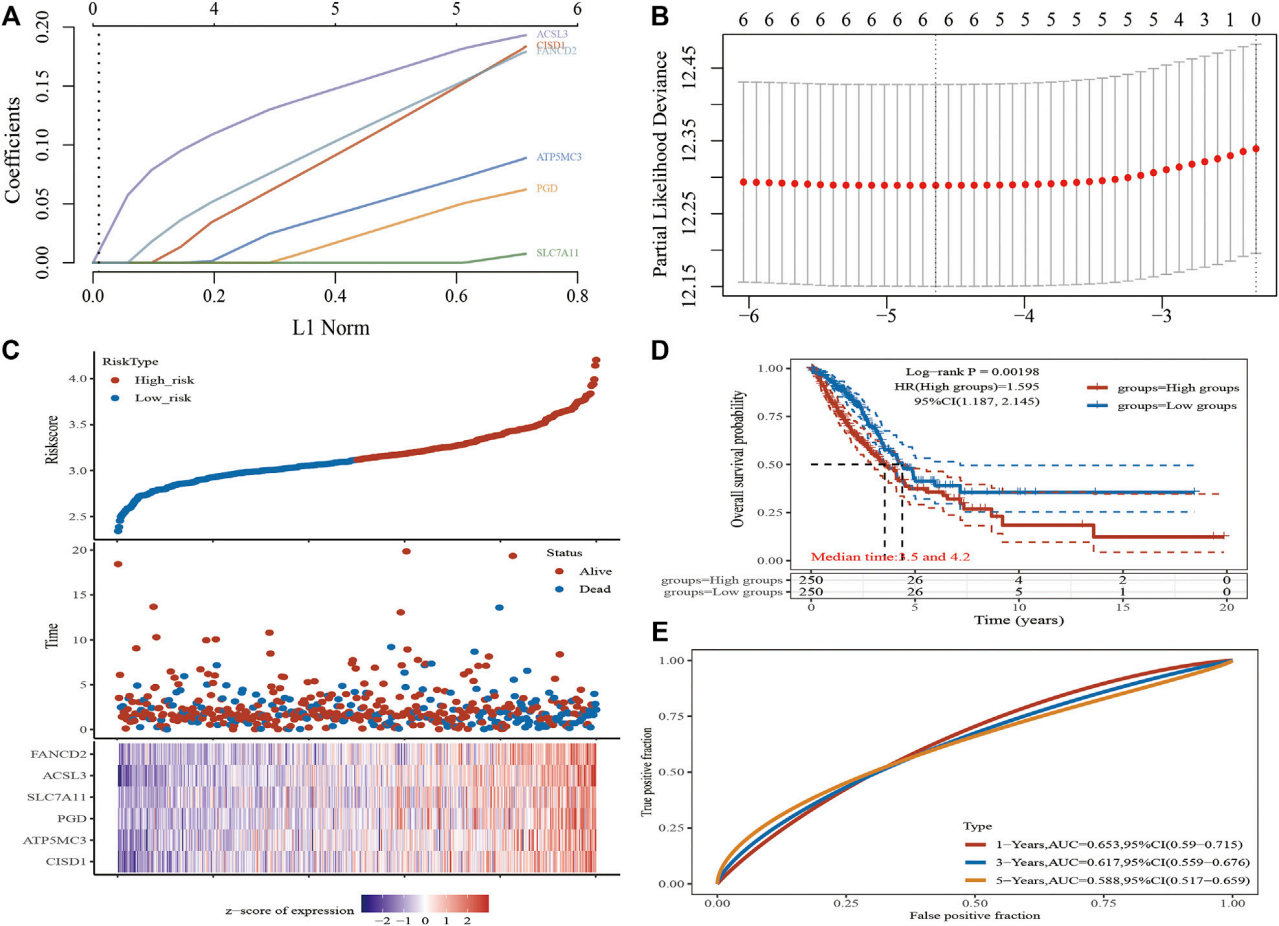
Construction of a prognostic FRG model in LUAD. **(A)** LASSO coefficient profiles of six FRGs. **(B)** Plots of the ten-fold cross-validation error rates. **(C)** Distribution of risk score, survival status, and the expression of six prognostics FRGs in LUAD. **(D,E)** Overall survival curves for LUAD patients in the high-/low-risk group and the ROC curve of measuring the predictive value. *p < 0.05, **p < 0.01, ***p < 0.001.

**FIGURE 5:**
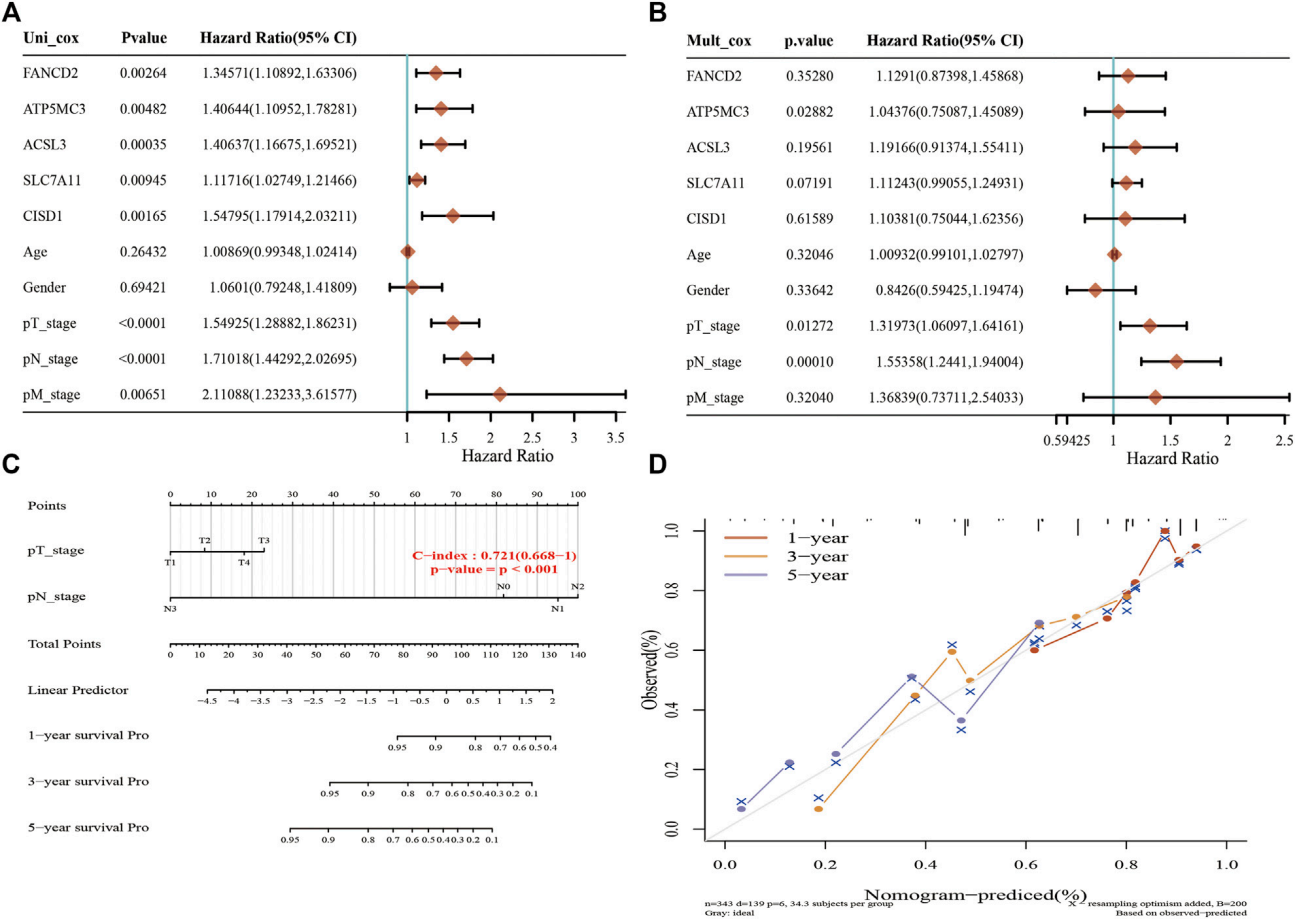
Construction of a predictive nomogram in LUAD. **(A,B)** Hazard ratio and *p*‐value of the constituents involved in univariate and multivariate Cox regression considering clinical parameters and six prognostics FRGs in LUAD. **(C,D)** Nomogram predicts the 1-, 3-, and 5-year overall survival rate of LUAD patients and calibration curve for the overall survival nomogram model in the discovery group. A dashed diagonal line represents the ideal nomogram. *p < 0.05, **p < 0.01, ***p < 0.001.

A Lasso Cox regression prognostic model based on the six aforementioned FRGs was constructed (Figures 4A,B) with the risk score=(0.0387)*PGD+(0.082)*ATP5MC3+(0.2118)*CISD1+(0.0029)*SLC7A11+(0.2104)*ACSL3+(0.1528)*FANCD2. Patients suffering LUAD were divided into two groups based on the risk score. Risk score distribution, survival status, and gene expression data are presented in Figure 4C. Patient risk of death increased and survival time decreased as the risk score increased (Figure 4C). Kaplan–Meier survival analysis suggested that LUAD patients with high-risk scores possess a worse overall survival probability as compared to those with low-risk scores (median time = 3.3 vs. 4.2 years, *p* = 0.00198; Figure 4D), with AUCs of 0.653, 0.617, and 0.588 noted on 1-, 3-, and 5-year receiver operating characteristic (ROC) curves, respectively (Figure 4E). The above results were verified by the GEO dataset; Kaplan–Meier survival analysis suggested that LUAD patients with high-risk scores possess a worse overall survival probability as compared to those with low-risk scores (median time = 2.9 vs. 4.9 years, *p* = 6.52e-08), with AUCs of 0.66, 0.693, and 0.664 noted on 1-, 3-, and 5-year ROC curves, respectively (Supplementary Figures S6A–D). A nomogram predictive of patient survival state was constructed. Univariate and multivariate analyses revealed ATP5MC3 expression and TNM stage to be independent factors affecting LUAD prognosis (Figures 5A,B). The nomogram could accurately predict 1-, 3-, and 5‐year overall survival rates as compared with an ideal model of the entire cohort (Figures 5C,D).

### Analysis of the Correlation Between FRG Expression and TMB, MSI, and Drug Sensitivity

Emerging evidence has suggested that TMB and MSI may serve as potential predictive biomarkers for immunotherapy efficacy in the setting of lung cancer (Goodman et al., 2019). As such, several studies have reported that the expression of FRGs significantly correlates with tumor immune infiltration. Correlation analysis revealed FANCD2, ATP5MC3, and PGD expression to positively correlate with TMB (Supplementary Figure S3A); FANCD2 expression was also noted to positively correlate with MSI. Meanwhile, CISD1 expression was found to negatively correlate with MSI in LUAD (Supplementary Figure S3B). To explore potential therapeutic targets, gene-set co-expression analysis was utilized to explore the relationship between FRG expression and drug sensitivity. Results revealed SLC7A11 expression to positively correlate with sensitivity to PRIMA-1, PX-12, necrosulfonamide, methylstat, piperlongumine, SMER-3, NSC95397, manumycin A, ML162, PL-DI1S, 3R-RSL-3, pifithrin-mu, and cerulenin (*r* > 0.36). Expression of ACSL3 was found to positively correlate with sensitivity to tozasertib, PRIMA-1, PX-12, manumycin A, BRD-K30748066, ML210, BRD-A94377914, 1S, 3R-RSL-3, and docetaxel (*r* > 0.30). On the contrary, PGD expression was found to negatively correlate with sensitivity to tivantinib, SCH-79797, BI-2536, GW-405833, GSK461364, nakiterpiosin, docetaxel, SB-743921, bafilomycin A1, linifanib, ceranib-2, BRD-K70511574, BRD-K01737880, FQI-2, and BRD-K30748066 (*r* < -0.22). Expression of ATP5MC3 was found to negatively correlate with sensitivity to BRD-K30748066, marinopyrrole A, COL-3, dinaciclib, BI-2536, alvocidib, methotrexate, and bafilomycin A1 (*r* < -0.22). The expression of FANCD2 was found to negatively correlate with sensitivity to GSK-J4, BRD-K30748066, COL-3, docetaxel, GSK461364, BRD-K66453893, tivantinib, BI-2536, narciclasine, BRD-K70511574, and triazolothiadiazine (*r* < -0.34; Supplementary Figure S3D). These findings thus underscore that FRG expression either positively or negatively correlates with LUAD sensitivity to drugs.

### FRG Expression Associated With LUAD Immune Infiltration

**FIGURE 6:**
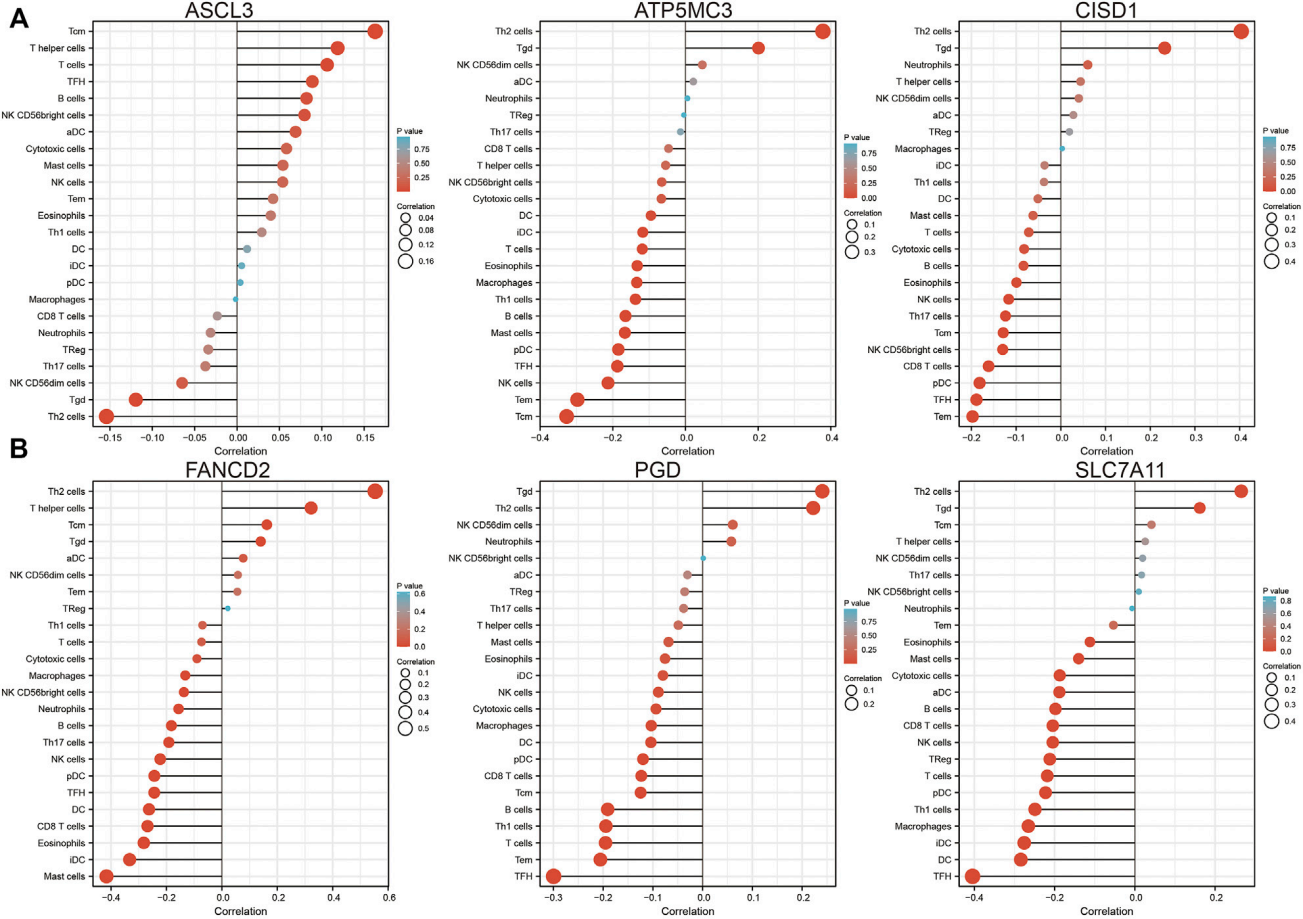
Analysis of the association between FRG expression and immune infiltration level in LUAD. **(A,B)** The association between FRG expression and immune infiltration level in LUAD.

Analysis of CISD1, FANCD2, PGD, ASCL1, ATP5MC3, and SLC7A11 expression in the setting of C1 and C2 LUAD subtypes revealed high CISD1, FANCD2, and ATP5MC3 expression in the C2 subtype and high PGD and SLC7A11 expression in the C1 subtype (Supplementary Figures S4A,B). Analysis of data obtained from the TIMER database revealed FRG somatic copy number alterations to significantly correlate with levels of immune cell infiltration in LUAD (Supplementary Figures S5A–F). As ferroptosis plays crucial roles in both immunity and pulmonary carcinogenesis (Huang et al., 2021), the correlation between CISD1, FANCD2, PGD, ASCL3, ATP5MC3, and SLC7A11 expression and tumor immune infiltration in LUAD was evaluated utilizing data obtained from the TIMER database. Expression of ASCL3 was found to positively correlate with the level of infiltration by central memory T cells, T helper cells, T follicular helper cells, B cells, natural killer cells, CD56 bright natural killer cells, activated dendritic cells, cytotoxic cells, and mast cells. On the contrary, ATP5MC3 expression was found to negatively correlate with the level of infiltration by cytotoxic cells, dendritic cells, interstitial dendritic cells, T cells, eosinophils, macrophages, T helper one cells, B cells, mast cells, plasmacytoid dendritic cells, T follicular helper cells, natural killer cells, and central and effector memory T cells. The expression of CISD1 was found to negatively correlate with the level of infiltration by mast cells, T cells, cytotoxic cells, B cells, eosinophils, natural killer cells, T helper 17 cells, CD56 bright natural killer cells, CD8 T cells, plasmacytoid dendritic cells, T follicular helper cells, and central and effector memory T cells (Figure 6A). The expression of FANCD2 was found to negatively correlate with the level of infiltration by cytotoxic cells, macrophages, CD56 bright natural killer cells, neutrophils, B cells, T helper 17 cells, natural killer cells, plasmacytoid dendritic cells, T follicular helper cells, dendritic cells, CD8 T cells, eosinophils, interstitial dendritic cells, and mast cells. Similarly, the PGD expression was found to negatively correlate with the level of infiltration by mast cells, eosinophils, interstitial dendritic cells, natural killer cells, cytotoxic cells, macrophages, dendritic cells, plasmacytoid dendritic cells, CD8 T cells, B cells, T helper one cell, T cells, T follicular helper cells, and effector and memory T cells. The expression of SLC7A11 was found to negatively correlate with the level of infiltration by eosinophils, mast cells, cytotoxic cells, activated dendritic cells, B cells, CD8 T cells, natural killer cells, regulatory T cells, T cells, plasmacytoid dendritic cells, T helper one cell, macrophages, interstitial dendritic cells, dendritic cells, and T follicular helper cells (Figure 6B).

**FIGURE 7:**
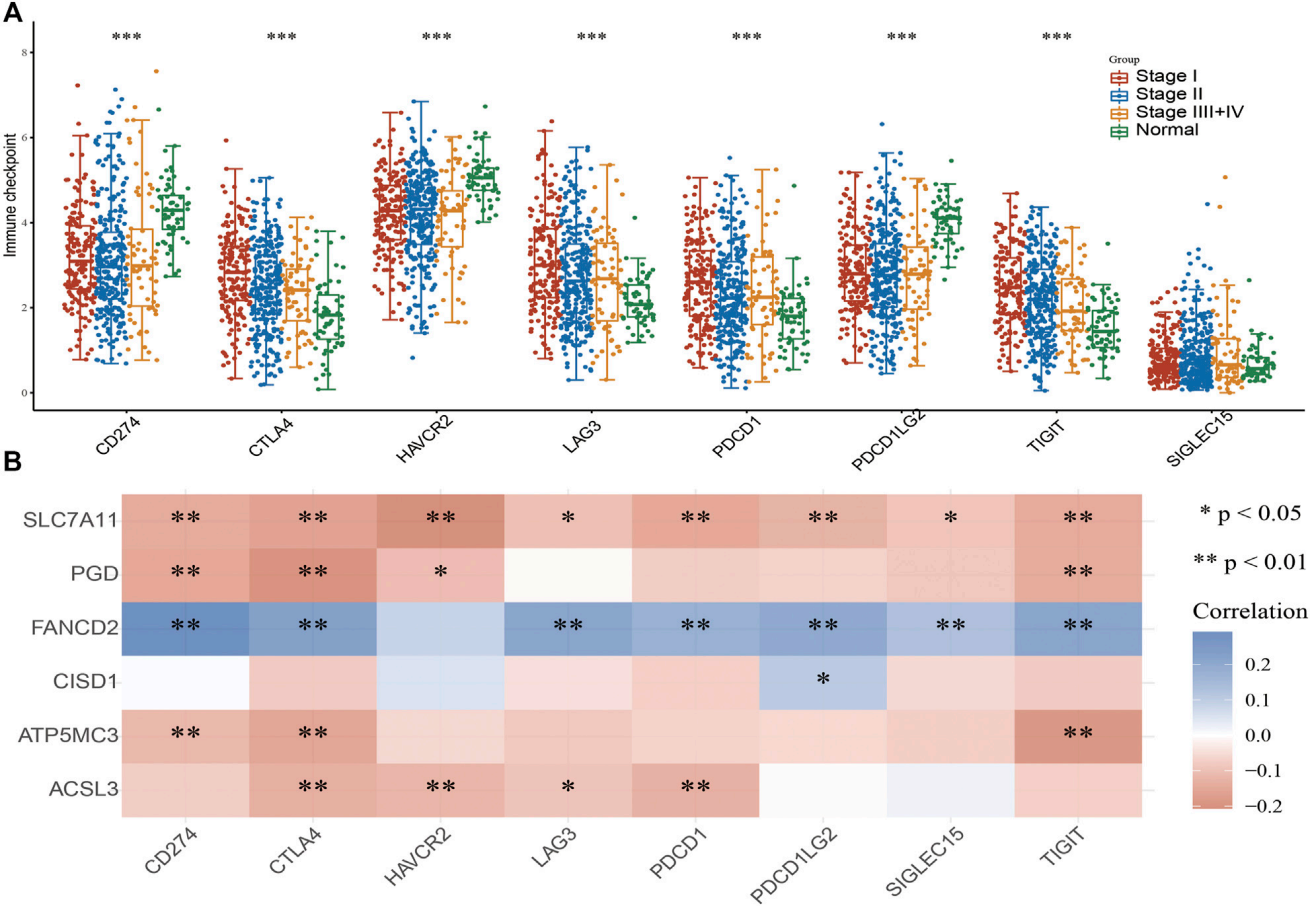
Analysis of the correlation between FRG expression and diverse immune modulators. **(A)** The expression of immune checkpoint-related genes in diverse pathologic stages and normal samples. **(B)** The correlation between FRGs expression and diverse immune checkpoint-related genes in LUAD. For all figures, ∗, ∗∗, and ∗∗∗ indicate *p* < 0.05, *p* < 0.01, and *p* < 0.001, respectively.

As immune checkpoints play a crucial role in tumor immunosuppression, we analyzed the distribution of a relevant gene expression in stage I–IV LUAD tissues (Figure 7A). We additionally evaluated the relationship between FRG and immune checkpoint-related gene (including CD274, CTLA4, HAVCR2, LAG3, PDCD1, PDCD1LG12, TIGIT, SIGLEC15) expression in LUAD *via* Pearson correlation analysis; results revealed the expression of SLC7A11, PGD, CISD1, ATP5MC3, and ACSL3 to negatively correlate with that of CD274, CTLA4, HAVCR2, LAG3, PDCD1, PDCD1LG12, TIGIT, and SIGLEC15. Meanwhile, FANCD2 expression was found to positively correlate with checkpoint-related gene expression (Figure 7B). These findings confirm that FRG expression significantly correlates with that of immune checkpoint-related genes in LUAD.

### Analysis of the Upstream FRG Molecular Regulatory Axis

**FIGURE 8:**
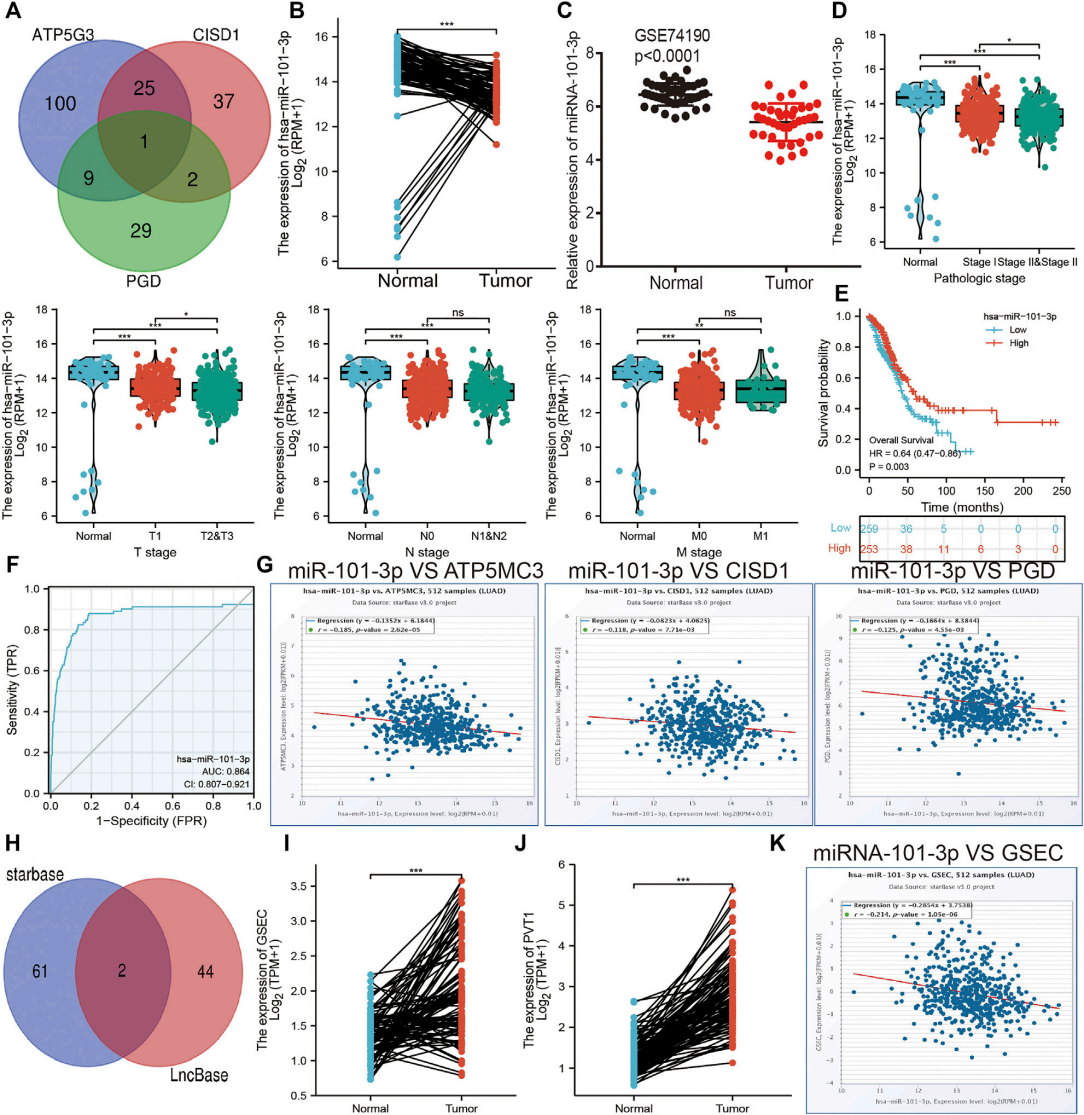
Prediction and analysis of the upstream miRNAs of FRGs in LUAD. **(A)** Prediction of the potential miRNAs of FRGs in LUAD examined by starbase. **(B,C)** The expression of miRNA-101-3p in LUAD was examined by TCGA/GEO databases. **(D)** The correlation between FRGs and clinical features in LUAD. **(E)** The prognosis value of miRNA-101-3p in LUAD. **(F)** ROC curve analyses and AUC values for miRNA-101-3p in LUAD. **(G)** Pearson’s correlation analysis determined the correlation between miRNA-101-3p and ATP5MC3, CISD1, and PGD expression in LUAD examined by starbase. **(H)** Explored the potential lncRNAs of miRNA-101-3p in LUAD by starbase. **(I,J)** The expression of GESC and PVT1 in LUAD was examined by starbase. **(K)** Pearson’s correlation analysis determined the correlation between miRNA-101-3p and GSEC in LUAD. *p < 0.05, **p < 0.01, ***p < 0.001.

**FIGURE 9:**
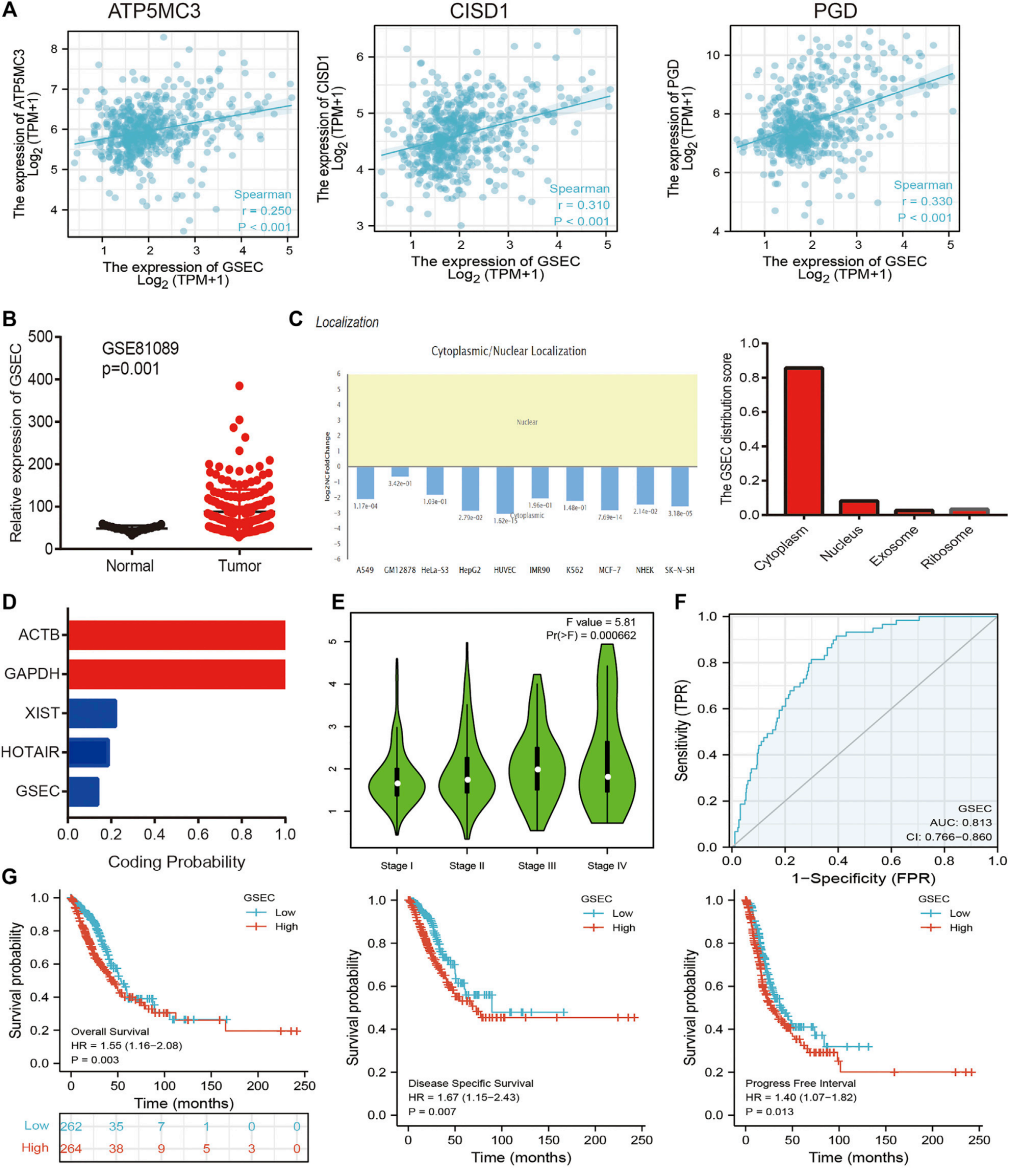
Construction of lncRNA/miRNA/FRG interaction network. **(A)** Pearson’s correlation analysis determined the correlation between GSEC and ATP5MC3, CISD1, and PGD in LUAD. **(B)** The expression of GESC in LUAD is examined by the GEO dataset. **(C)** The subcellular location of GESC is examined by lncLocator and Annolnc2 databases. **(D)** The coding ability of GESC is examined by coding potential calculator databases. **(E)** The pathologic stage of GESC in LUAD is examined by GEPIA databases. **(F)** ROC curve analyses and AUC values for GSEC in LUAD. **(G)** The prognosis of GESC in LUAD is examined by GEPIA databases. *p < 0.05, **p < 0.01, ***p < 0.001.

The aforementioned evidence suggests that ATP5MC3, PGD, and CISD1 expression correlates with both stage and progression of LUAD. To further explore the relevant upstream FRG regulatory axis, data obtained from various public databases were used to construct a network of mRNA–miRNA–lncRNA interactions. We found miRNA-101-3p to most significantly bind the 3′UTR of ATP5MC3, PGD, and CISD1 (Figure 8A). Further analysis revealed miRNA-101-3p to be decreased in lung cancer based on TCGA-LUAD and GSE74190 datasets (Figures 8B,C). Moreover, downregulated miRNA-101-3p expression correlated with the manifestation of poorer clinical features and prognosis among lung cancer patients (Figures 8D,E). Analysis of miRNA-101-3p ROC curve data revealed an AUC value of 0.864 among lung cancer patients (Figure 8F). We also found miRNA-101-3p expression to significantly negatively correlate with ATP5MC3, PGD, and CISD1 expression in LUAD (Figure 8G). To predict and obtain the PVT1 and GSEC lncRNAs, starbase and lncbase were utilized (Figures 8H–J). According to the ceRNA theory, lncRNA negatively and positively correlates with miRNA and mRNA expression, respectively. We found only GSEC to negatively correlate with miRNA-101-3p expression (Figure 8K) and positively correlate with ATP5MC3, PGD, and CISD1 expression in LUAD (Figure 9A). Importantly, GSEC was found to be highly expressed in the setting of this malignancy based on the GSE81089 dataset (Figure 9B) and primarily localized to the cytoplasm as determined using the lncLocator and AnnoLnc RNA tools (Figure 9C). However, GSEC was not found to possess coding potential (Figure 9D). High levels of GSEC expression were found to correlate not only with tumor stage but also with poor prognosis in LUAD. Analysis of the GSEC ROC curve data revealed an AUC value of 0.813 in LUAD patients (Figures 9E–G). In summary, our findings confirm that the lncRNA GSEC/miRNA-101-3p axis modulates ATP5MC3, PGD, and CISD1 expression in LUAD.

### Knockdown of CISD1 Inhibits LUAD Proliferation and Migration

**FIGURE 10:**
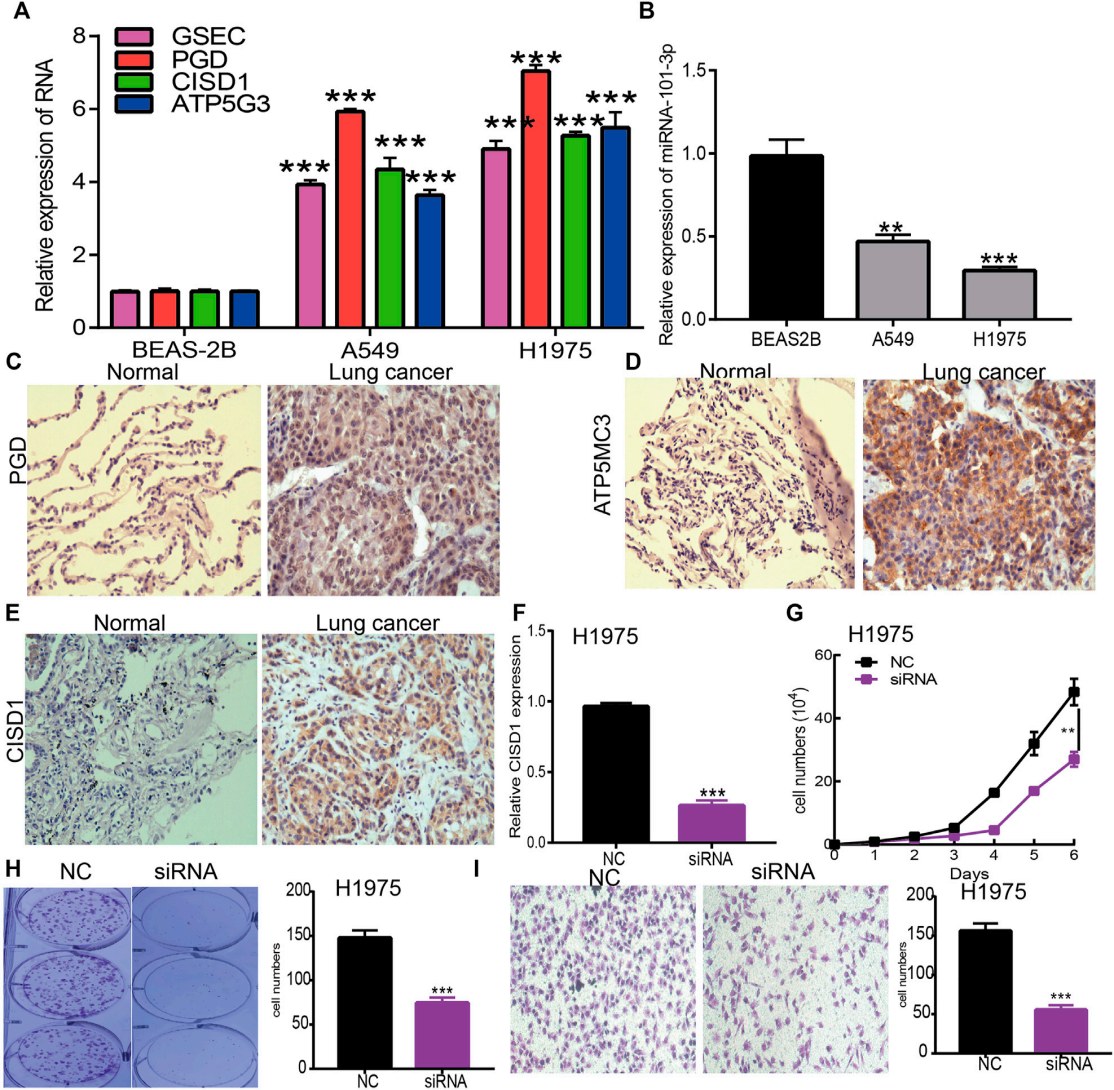
Depletion of CISD1 inhibits LUAD cell proliferation and migration. **(A,B)** The expression of GSEC, FRGs, and miRNA-101-3p in LUAD cell lines was examined by qRT-PCR assay. **(C–E)** The expression of PGD, ATP5MC3, and CISD1 in LUAD tissue was examined by IHC assay. **(F)** The expression of CISD1 in H1975 cells after knockdown of CISD1 was examined by qRT-PCR assay. **(G,H)** The growth curve and colony formation assays were utilized to detect the silencing of CISD1 on the growth of H1975 cells. **(I)** The transwell assay was utilized to detect the silencing of CISD1 on the migration of H1975 cells. *p < 0.05, **p < 0.01, ***p < 0.001.

To verify the aforementioned findings, a qRT-PCR assay was used to detect GSEC, ATP5MC3, PGD, CISD1, and miRNA-101-3p expression in LUAD cell lines. While we found GSEC, ATP5MC3, PGD, and CISD1 expression to be upregulated in LUAD cells (Figure 10A), miRNA-101-3p expression was noted to be decreased (Figure 10B). The immunohistochemical evaluation further revealed PGD, ATP5MC3, and CISD1 overexpression in LUAD tissues (Figures 10C–E). As prior literature has detailed the roles of PGD and ATP5MC3 in LUAD, we focused on further studying the influence of CISD1 on LUAD cell proliferation and migration *via* the utilization of target siRNA transfected into H1975 cells. We found that CISD1 expression was decreased after CISD1 knockdown in this cell line (Figure 10F). Growth curve and colony formation findings revealed that CISD1 knockdown significantly inhibits cell proliferation (Figures 10G,H). Transwell assays indicated that the migratory capability of H1975 cells was significantly suppressed *via* CISD1 downregulation (Figure 10I). These findings confirm that CISD1 functions as an oncogene, promoting cell growth and migration among LUAD cells.

## Discussion

An iron-dependent type of programmed cell death, ferroptosis differs from other forms of cell death such as apoptosis, necrosis, and autophagy. The ferroptosis process generally results in excessive lipid peroxidation and is induced by abnormalities in cellular redox processes. Importantly, ferroptosis is understood to play an indispensable role in the elimination of carcinogenic cells (Hirschhorn and Stockwell, 2019). Activation of Ras/mitogen-activated protein kinase signaling increases the sensitivity of cancer cells to ferroptosis and thus leads to the maintenance of relative iron abundance in cancer *via* control of transferrin receptor and ferritin expression (Dixon et al., 2012). Knockdown of FMS-like tyrosine kinase three was reported to reduce lipid peroxidation and inhibit p22phox activity, resulting in the inhibition of ferroptosis among malignant cells (Song et al., 2018). Recently, 5-monophosphate was reported to increase Beclin1 phosphorylation and inhibit system Xc activity, thus facilitating ferroptosis (Kang et al., 2018). However, the modulatory mechanism of the ferroptosis process and associated FRG expression correlated with LUAD progression remains to be elucidated.

Here, we first investigated the expression and prognostic value of FRGs in LUAD. We found a total of 26 genes to be upregulated; seven genes were found to be downregulated in LUAD as compared with normal tissue. Meanwhile, the expression of FDFT1, IREB2, NFS1, TFRC, HSBP1, ACO1, LPCAT3, CARS, GSS, ACACA, ACSL3, CISD1, SLC1A5, and NCOA4 was found to positively correlate with respective CNV in LUAD. Levels of DNA methylation were found to negatively associate with CBS, FDFT1, STEAP3, FADS2, SCL1A5, GCLM, GOT1, SLC7A11, CD44, ACSL3, and G6PD expression in LUAD. Collectively, these data indicate that CNV and DNA methylation exert critical effects on FRG expression in LUAD.

Functional enrichment analysis revealed 33 FRGs to be primarily involved in vascular fluid dynamics and atherosclerosis, HIF-1a signaling, lipid metabolism, ferroptosis, cysteine and methionine metabolism, glutathione metabolism, amino acid biosynthesis, necroptosis, arachidonic acid metabolism, thyroid hormone synthesis, serotonergic communication, alanine metabolism, aspartate metabolism, glutamate metabolism, inflammatory bowel disease, 2-monocarboxylic acid metabolism, and arginine biosynthesis. Our findings demonstrate that FRGs play an important role in both LUAD pathogenesis and illness progression.

Prognosis analysis revealed increased SLC7A11, PGD, FANCD2, CISD1, ATP5G3, and ASCL3 expression to closely correlate with poor LUAD clinical prognosis. Importantly, levels of ATP5G3, CISD1, and PGD expression were found to positively correlate with tumor stage. Analysis ROC curve data revealed SLC7A11, FANCD2, CISD1, and ATP5G3 to be highly accurate markers in LUAD (AUC > 0.8). These findings suggest that FRG expression correlates with LUAD prognosis. A highly accurate five-gene prognostic model capable of predicting overall LUAD patient survival was constructed based on SLC7A11, PGD, FANCD2, SIDS1, ATP5G3, and ASCL3 using Lasso Cox regression analysis. A predictive nomogram useful in predicting 3- and 5‐year overall survival rates as compared to an ideal model in the studied cohort was similarly developed. Here, we identified a pyroptosis-related prognostic gene signature for LUAD, further advancing potential prognostic methods relevant to LUAD.

Here, SLC7A11, PGD, FANCD2, CIDS1, ATP5G3, and ASCL3 were found to serve as effective gene signatures for predicting LUAD prognosis. An important cystine/glutamate antiporter regulated by p53, SLC7A11 plays suppressive roles in the ferroptosis process. The SLC7A11-mediated importation of cystine enhances glutathione biosynthesis and increases GPX4-mediated detoxification of lipid peroxides, thereby inhibiting ferroptosis (Stockwell and Jiang, 2020). A prior study reported PGD capable of predicting overall survival in the setting of papillary thyroid carcinoma (Yang et al., 2021). In addition, FANCD2 was reported to inhibit ferroptosis and protect from bone marrow injury (Song et al., 2016).

Importantly, we found FRG expression to significantly correlate with tumor immune infiltration. Furthermore, we found that SLC7A11, PGD, CISD1, ATP5MC3, and ACSL3 expression significantly negatively correlates with CD274, CTLA4, HAVCR2, LAG3, PDCD1, PDCD1LG12, TIGIT, and SIGLEC15 expression. However, FANCD2 expression was found to positively correlate with checkpoint-related gene expression in LUAD. As such, these data confirm that FRG expression is associated with immune checkpoint-related gene expression in LUAD.

**FIGURE 11:**
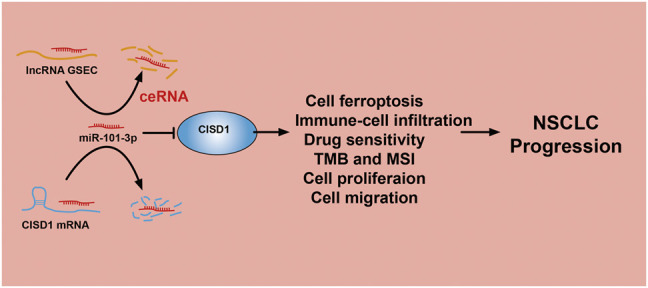
A working model for lncRNA GSEC/miR-101-3p/CISD1 axis in LUAD.

The lncRNA GSEC was previously reported to upregulate EIF5A2 expression *via* miR-588 sponging and subsequently facilitate cell proliferation, migration, and invasion capabilities in osteosarcoma (Liu et al., 2020). High levels of GSEC expression were reported in triple-negative breast cancer tissue and cell lines, with GSEC knockdown significantly decreasing these proliferative, migratory, and invasive capabilities. The sponging of miR-202-5p by GSEC, along with resultant increased AXL expression, was reported to enhance LUAD progression (Zhang et al., 2021). Similarly, miRNA-101-3p was reported to inhibit both proliferative and migratory capabilities of gastric carcinoma cells and enhance apoptosis *via* PIM-1 expression modulation (Wu et al., 2019). Indeed, in an LUAD setting of miRNA-101-3p downregulation, overexpression of miR-101-3p mimics was reported to reduce rates of tumor growth and progression *in vitro* (Liu et al., 2021). In this study, data obtained from various public databases were used to illustrate novel mRNA–miRNA–lncRNA interactions (namely, the lncRNA GSEC/miR-101-3p/ATP5MC3/PGD/CISD1 axis) detailing modulation of FRG expression following the ceRNA hypothesis (Figure 11). Although we found GSEC, ATP5MC3, PGD, and CISD1 to be upregulated in LUAD cell lines, miRNA-101-3p expression was found to be decreased. The immunohistochemical evaluation also confirmed increased expression of ATP5MC3, PGD, and CISD1 in LUAD tissue. Knockdown of CISD1 was found to significantly inhibit the proliferative and migratory capabilities of LUAD cells.

## Conclusion

Here, we found that GSEC, CISD1, ATP5MC3, and PGD are upregulated, and miRNA-101-3p is downregulated, in LUAD. Knockdown of CISD1 was found to significantly inhibit the proliferative and migratory capabilities of LUAD cells. In summary, this study characterizes the functional roles of the lncRNA GSEC/miR-101-3p/CISD1 axis in LUAD and suggests potential diagnostic and therapeutic biomarkers for future clinical application in the management of this illness.

## Data Availability

The original contributions presented in the study are included in the article/Supplementary Material; further inquiries can be directed to the corresponding author.
