# Supplementary material for: Systematic Analysis and Validation of the Prognosis, Immunological Role and Biology Function of the Ferroptosis-Related lncRNA GSEC/miRNA-101-3p/CISD1 Axis in Lung Adenocarcinoma
Source: Front Mol Biosci. 2022 Mar 7;8:793732. doi: 10.3389/fmolb.2021.793732 (PMC8936422; doi:10.3389/fmolb.2021.793732)
Supplement: Supplementary file 1 [file DataSheet1.docx]

Supplementary Material

**
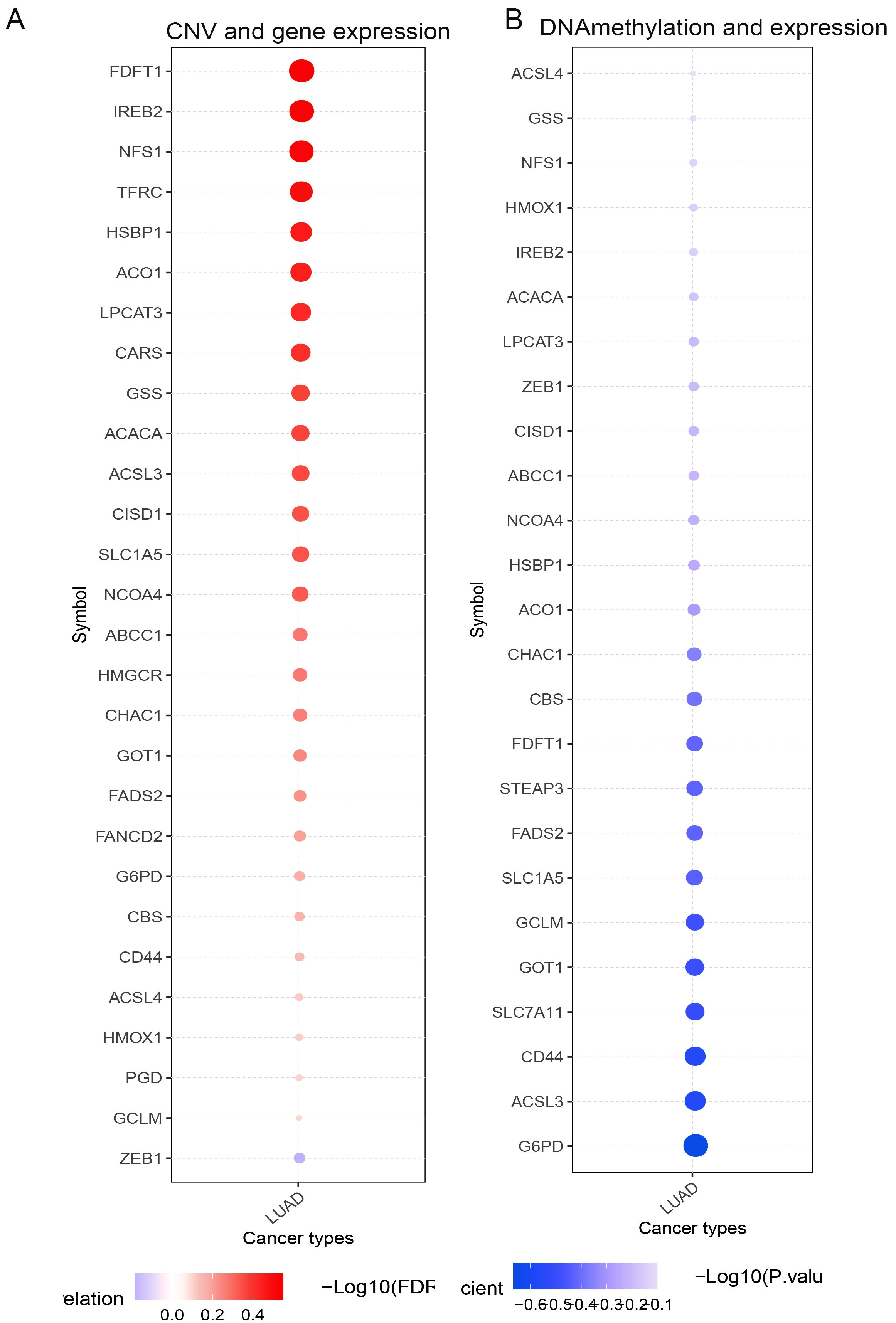
**

**Supplementary FIGURE 1 ⎜** **The correlation between CNV, DNA methylation and FRGs expression in NSCLC.**

(A) The correlation between CNV and FRGs expression in NSCLC. (B) The correlation between DNA methylation and FRGs expression in NSCLC.

**
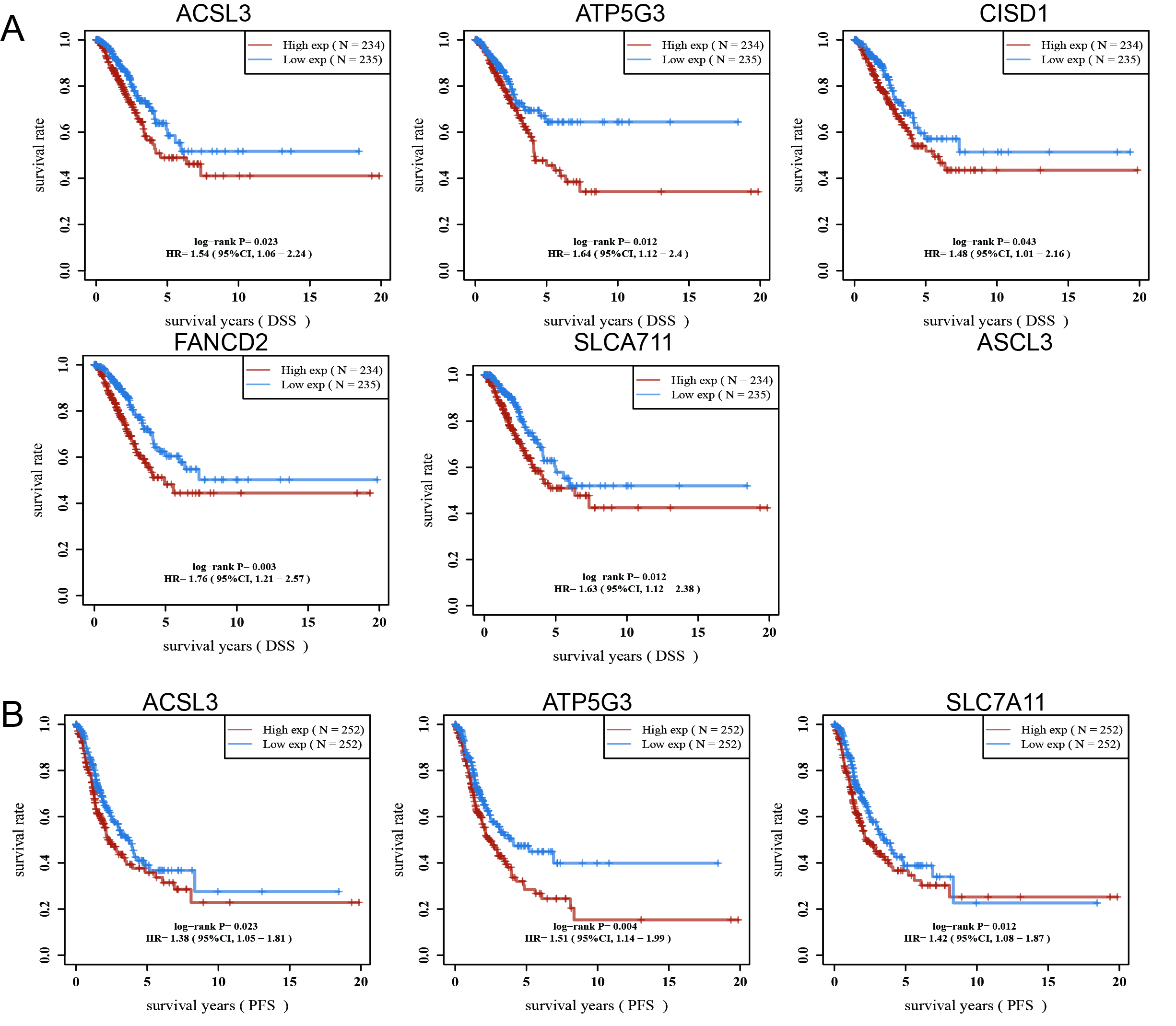
**

**Supplementary FIGURE 2 ⎜ The prognosis of FRGs in NSCLC.**

(A) The DSS of FRGs in NSCLC. (B) The PFS of FRGs in NSCLC.

**
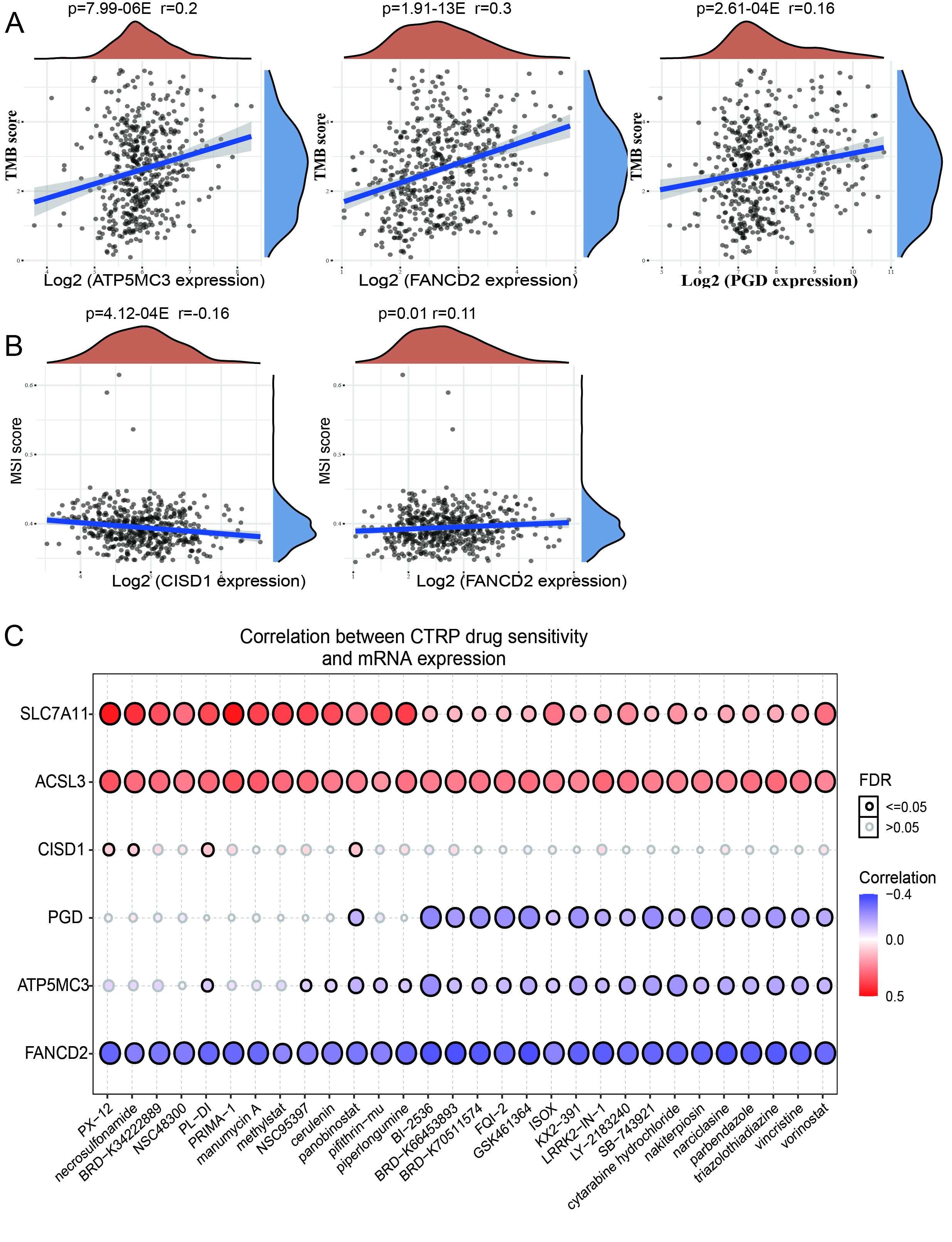
**

**Supplementary FIGURE 3 ⎜** **Analysis the correlation between the FRGs expression and TMB, MSI and drug sensitivity.** (A) The correlation between the FRGs expression and TMB in NSCLC. (B) The correlation between the FRGs expression and MSI in NSCLC. (C) The correlation between the FRGs expression and drug sensitivity.


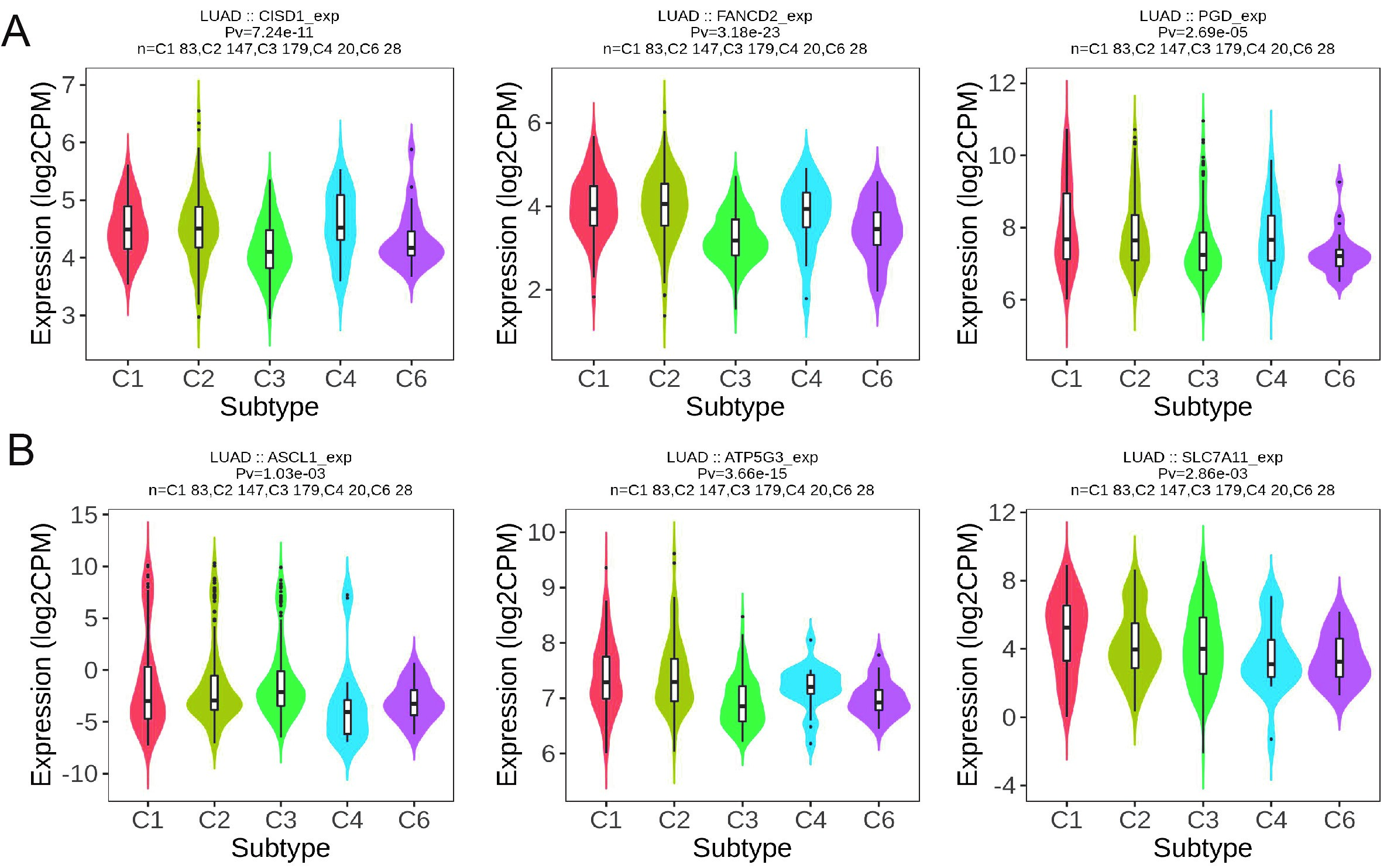


**Supplementary FIGURE 4 ⎜ The expression of FRGs in immune subtype of LUAD.**

(A-B) The expression of FRGs in immune subtype of NSCLC.


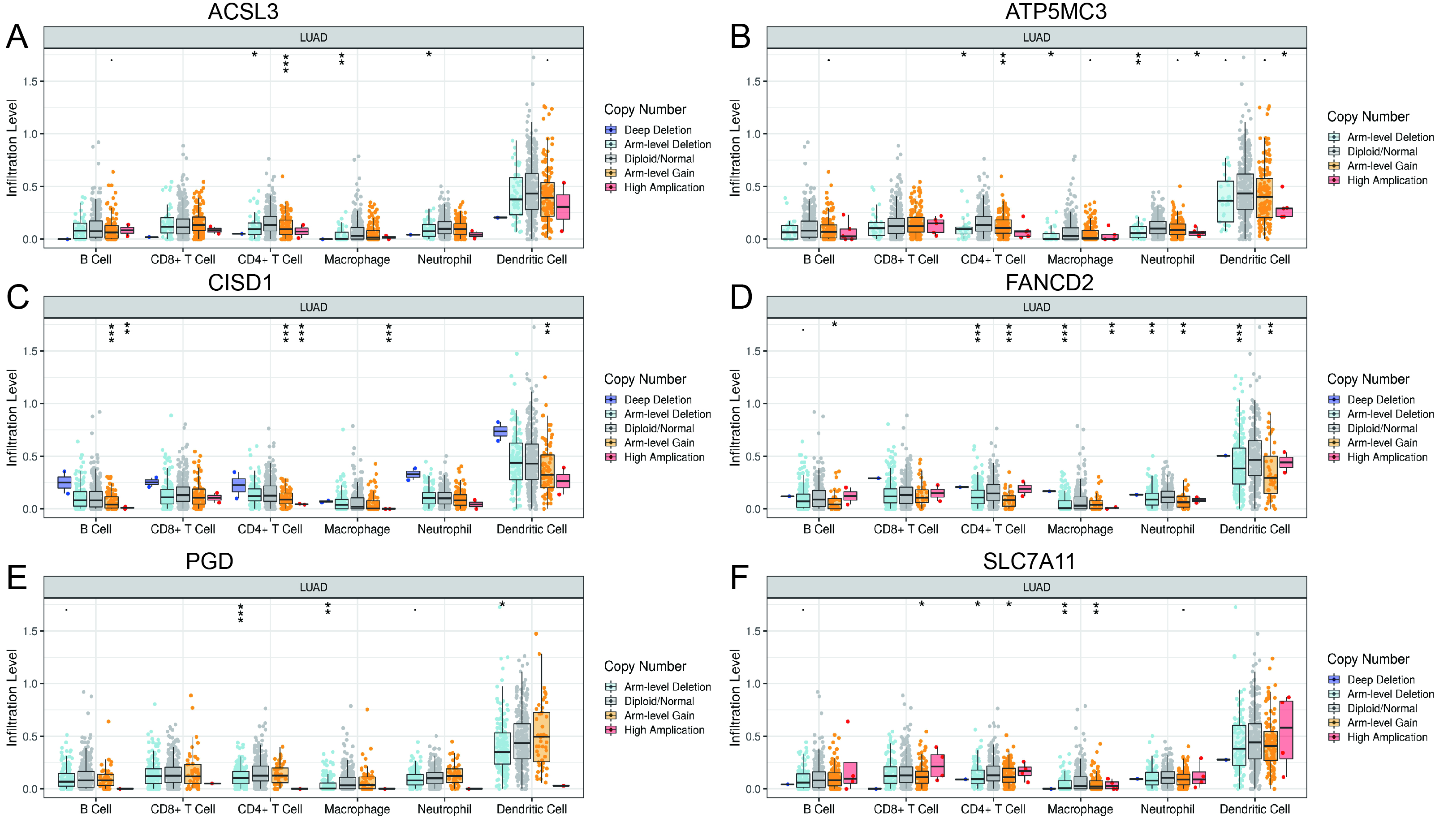


**Supplementary FIGURE 5 ⎜** **Analysis the correlation between the tumor infiltrating levels in NSCLC and somatic copy number alterations of FRGs.**

(A-F) the correlation between the tumor infiltrating levels and somatic copy number alterations of ACSL3 (A), ATP5MC3 (B), CISD1 (C), FANCD2 (D), PGD (E) and SLC7A11(F) in NSCLC.


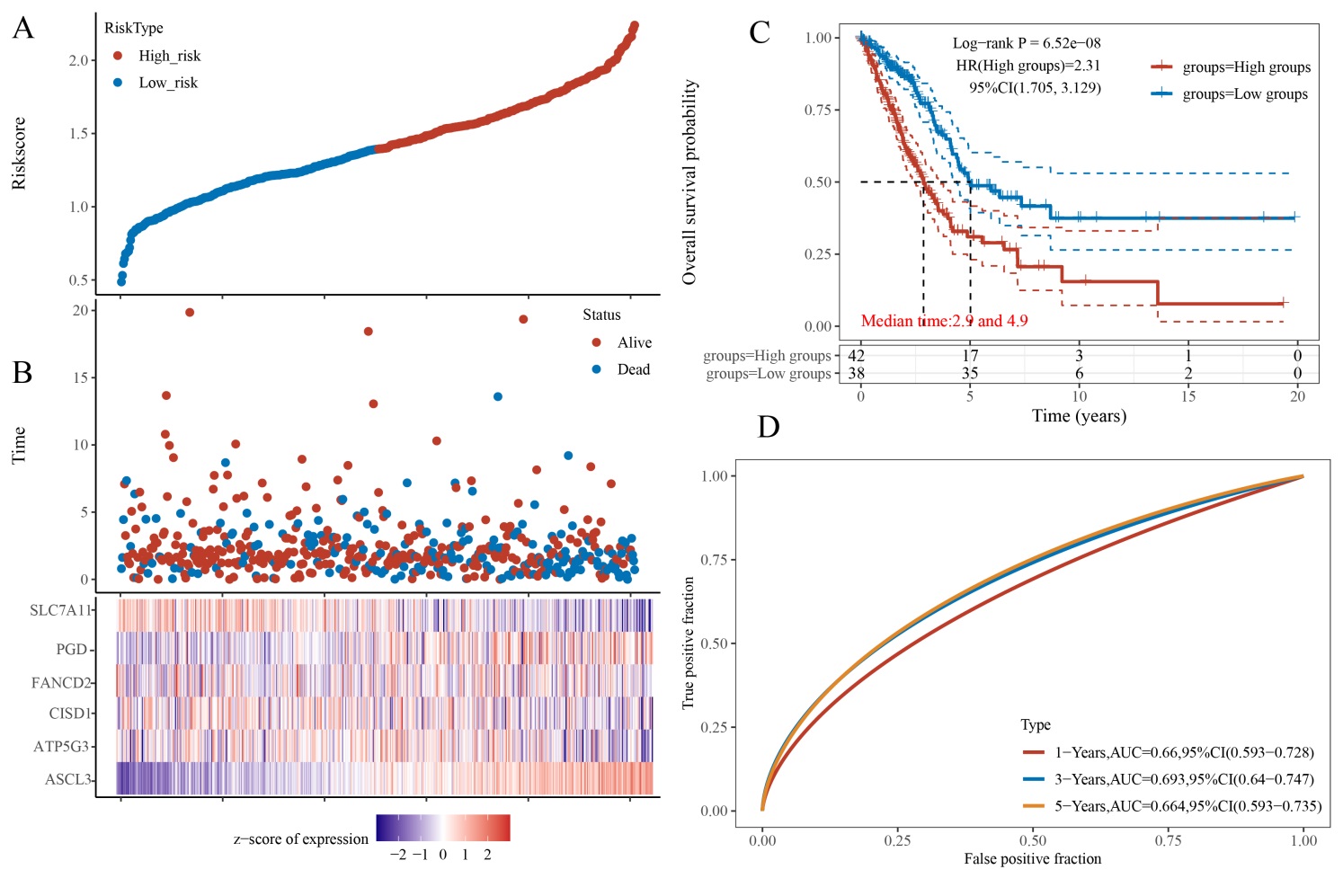


**Supplementary FIGURE 6 ⎜** **Construction of a prognostic FRG model in LUAD by GSE81089 dataset.**

(A-B) Distribution of risk score, survival status, and the expression of six prognostics FRGs in LUAD. (C-D) Overall survival curves for LUAD patients in the high-/low-risk group and the ROC curve of measuring the predictive value. *p < 0.05, **p < 0.01, ***p < 0.001

**Supplementary table 1. The FRGs gene list used in this manuscript.**

| FANCD2 | FDFT1 | HMGCR | LPCAT3 |
| --- | --- | --- | --- |
| TFRC | GSS | ABCC1 | GOT1 |
| IREB2 | FADS2 | HFE2L2 | HSBP1 |
| ACACA | NFS1 | CARS1 | STEAP3 |
| ACSL3 | ZEB1 | PGD | PTGS3 |
| GCLM | ATP5MC3 | ACSL4 | CISD1 |
| EMC2 | ACO1 | NCOA4 | CD44 |
| G6PD | CBS | SLC7A11 | CHAC1 |
| SLC1A5 | HMOX1 |  |  |
